# Identification of differentially expressed miRNAs and miRNA-targeted genes in bladder cancer

**DOI:** 10.18632/oncotarget.24441

**Published:** 2018-02-07

**Authors:** Jong-Young Lee, Seok Joong Yun, Pildu Jeong, Xuan-Mei Piao, Ye-Hwan Kim, Jihye Kim, Sathiyamoorthy Subramaniyam, Young Joon Byun, Ho Won Kang, Sung Phil Seo, Jayoung Kim, Jung Min Kim, Eun Sang Yoo, Isaac Y. Kim, Sung-Kwon Moon, Yung Hyun Choi, Wun-Jae Kim

**Affiliations:** ^1^ Department of Business Data Convergence, Chungbuk National University, Cheongju, Republic of Korea; ^2^ Department of Urology, College of Medicine, Chungbuk National University, Cheongju, Republic of Korea; ^3^ Department of Surgery, Department of Biomedical Sciences, Cedars-Sinai Medical Center, University of California Los Angeles, Los Angeles, California, USA; ^4^ NAR Center, Inc., Daejeon Oriental Hospital of Daejeon University, Daejeon, Republic of Korea; ^5^ Department of Urology, Kyungpook National University Hospital, Kyungpook National University School of Medicine, Daegu, Republic of Korea; ^6^ Section of Urologic Oncology and Dean and Betty Gallo Prostate Cancer Center, The Cancer Institute of New Jersey and Robert Wood Johnson Medical School, New Brunswick, New Jersey, USA; ^7^ Department of Food Science and Technology, Chung-Ang University, Ansung, Republic of Korea; ^8^ Department of Biochemistry, College of Oriental Medicine, Dong-Eui University, Busan, Republic of Korea; ^9^ Microarray Division, Theragen Etex Bio Institute, Suwon, Republic of Korea; ^10^ Microarray Division, SNP Medicine Co., Ltd, Suwon, Republic of Korea

**Keywords:** bladder cancer tumorigenesis, miRNA, mRNA, miRNA-mRNA interaction, transcription factor

## Abstract

**Background:**

Differentially expressed genes and their post-transcriptional regulator-microRNAs (miRNAs), are potential keys to pioneering cancer diagnosis and treatment. The aim of this study was to investigate how the miRNA-mRNA interactions might affect the tumorigenesis of bladder cancer (BC) and to identify specific miRNA and mRNA genetic markers in the two BC types: non-muscle invasive bladder cancer (NMIBC) and muscle invasive bladder cancer (MIBC).

**Results:**

We identified 227 genes that interacted with 54 miRNAs in NMIBC, and 14 genes that interacted with 10 miRNAs in MIBC. Based on this data, we found extracellular matrix-related genes are highly enriched in NMIBC while genes related to core nuclear division are highly enriched in MIBC. Furthermore, using a transcriptional regulatory element database, we found indirect regulatory transcription factors (TFs) for enriched genes could regulate tumorigenesis with or without miRNAs.

**Materials and methods:**

Tissue samples from 234 patients histologically diagnosed with BC and 83 individuals without BC were analyzed using microarray and next-generation sequencing technology, and we used different cut-offs to identify differentially expressed mRNAs and miRNAs in NMIBC and MIBC. The selected mRNAs and miRNAs were paired using validated target datasets and according to inverse expression relationships. MiRNA interacted genes were compared with the TF-regulating genes in BC. Meanwhile, pathway enrichment analysis was performed to identify the functions of selected miRNAs and genes.

**Conclusions:**

Identification of differential gene expression in specific tumor types could facilitate development of cancer diagnosis and aid in the early detection of BC.

## INTRODUCTION

Bladder cancer (BC) is the second most common urological malignancy in the United States. In 2015, approximately 73,510 new cases of BC were diagnosed and 14,680 individuals died of this disease in the U.S. [1]. In South Korea, BC is the second most common genitourinary tumor and the incidence of BC is about five times greater in men than it is in women [2].

Conventional diagnosis of BC classifies the tumor into two groups based on its clinico-pathologic features: non-muscle invasive (NMIBC) and muscle-invasive (MIBC). [3]. At the time of first diagnosis, a majority of cases are classified as NMIBC. However, approximately 20% of BC is confirmed as MIBC *in situ*, which is the main cause of cancer-specific deaths in BC patients. In general, NMIBC has a much better prognosis than MIBC and survival rates for NMIBC are higher than those for other malignancies. However, even after transurethral resection (TUR) of the primary tumor, the chance of recurrence is common with NMIBC. Approximately 30-50% of patients with NMIBC experience recurrence in their lifetime. NMIBC also runs the risk of possibly progressing into MIBC but the chances are usually low, ∼10-20% [4, 5]. Therefore, frequent recurrence and eventual progression to MIBC are challenges for both patients and urologists. Despite technical improvements in the diagnosis of BC, more efficient diagnostic tests that can facilitate early diagnosis and active surveillance are urgently needed. We currently lack an ideal non-invasive detection tool and there is a need for more accurate and predictive biomarkers.

To date, more than 1,100 microRNAs (miRNAs) have been identified in the human genome, far fewer than the total number of mRNAs. However, rather than being strictly one to one, interactions between miRNAs and target genes can be one-to-many or many-to-one, resulting in a very large number of potential regulatory effects. In addition, there is some controversy regarding the specific mode of how miRNA acts [6]. Several studies have shown that miRNAs repress mRNAs by regulating their stability or translation efficiency. Regarding BC, evidence from expression profiling studies using microarray-based approaches has provided abundant information to support the theory of miRNAs. Nevertheless, data about the interactions between miRNAs and mRNAs is inconsistent, and little is known about their expression levels and physiologically relevant functions in cancer cells.

In this study, we sought to identify NMIBC- or MIBC-specific miRNAs, predict their associated genes and miRNA-mRNA interactions based on bioinformatics analyses and validation by next-generation sequencing (NGS), and determine the critical networks and pathways that are dysregulated in BC patients. Our results provide important insight into the mechanisms underlying NMIBC and MIBC in human patients, as well as BC progression.

## RESULTS

### Identification of differentially expressed genes between BC tissue and normal tissue

**Figure 1:**
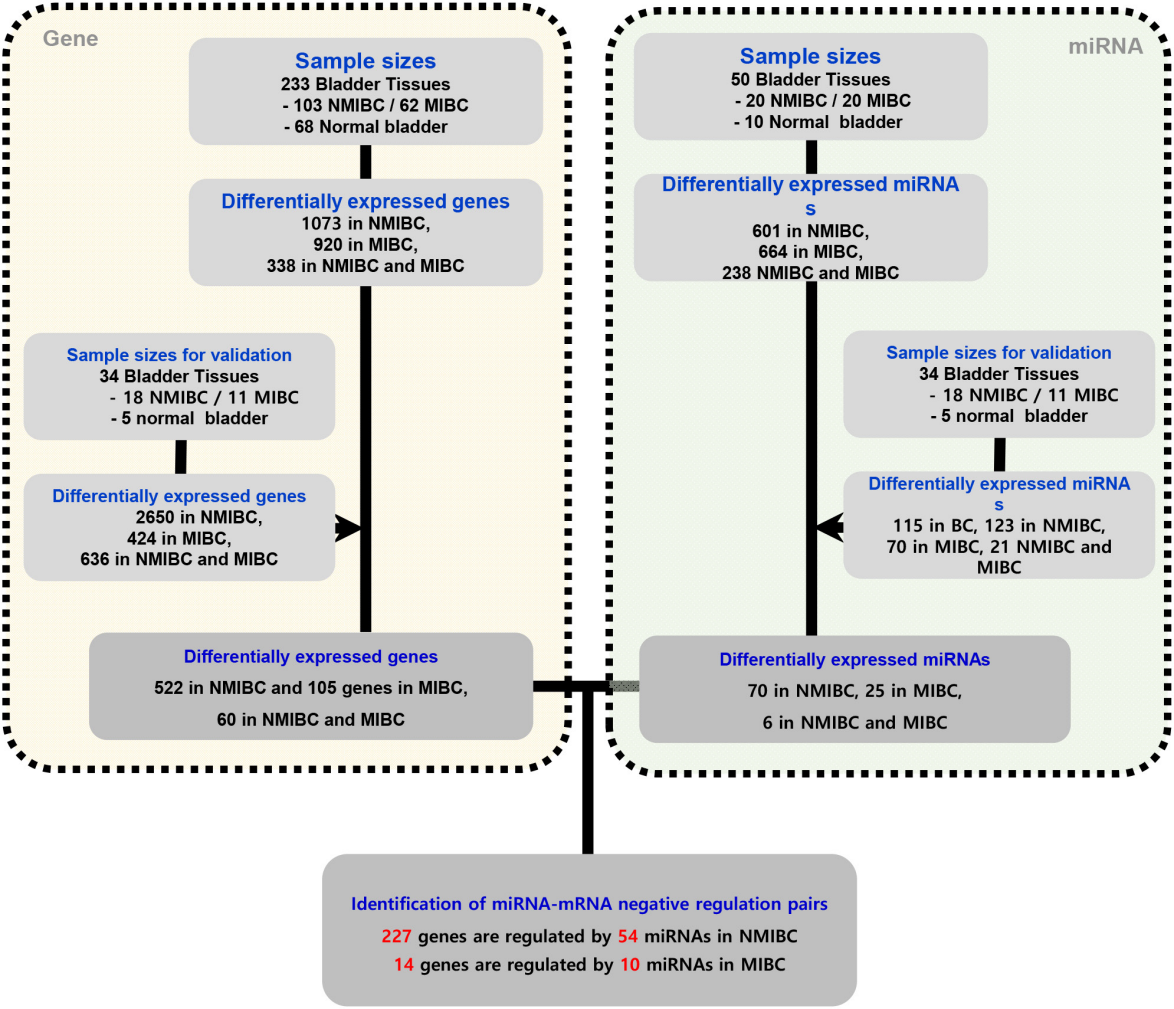
Schematic showing negatively correlated gene-miRNA pairs in NMIBC and MIBC Differentially expressed mRNAs and miRNAs were identified according to -fold changes in expression and *p*-value criteria. MiRNA-targeted gene pairs were identified using miRNAs and mRNAs differentially expressed in NMIBC and MIBC versus normal tissue according to previously validated informations regarding mRNA-miRNA interactions. NMIBC, non-muscle invasive bladder cancer; MIBC, muscle invasive bladder cancer.

**Figure 2:**
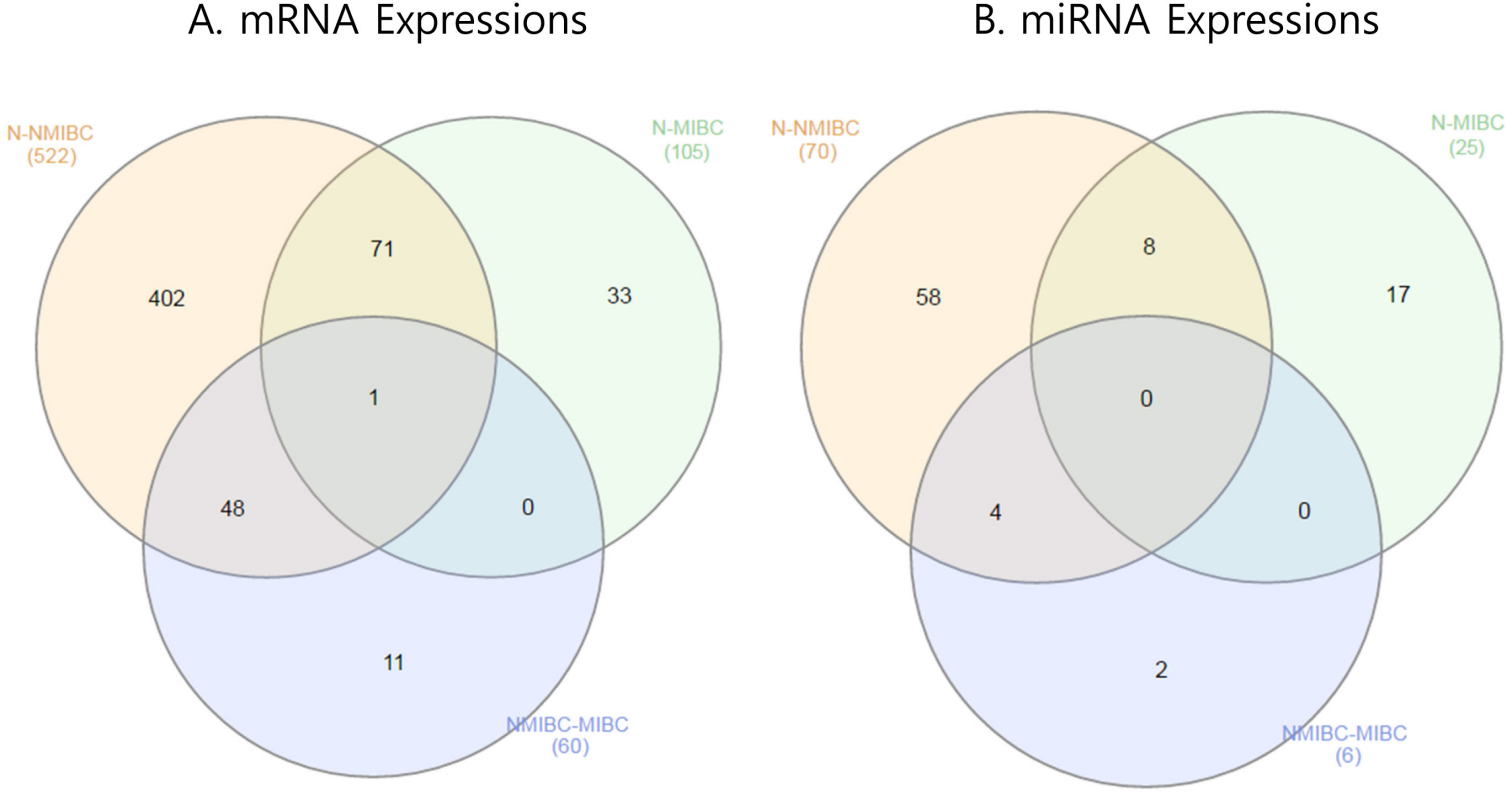
Venn diagram showing mRNAs and miRNAs differentially expressed in BC **(A)** mRNA expression. **(B)** miRNA expression. BC, bladder cancer; NMIBC, non-muscle invasive bladder cancer; MIBC, muscle invasive bladder cancer.

Determination of mRNAs was performed as shown in Figure 1. We extracted 522 genes that were differentially expressed in NMIBC versus normal tissue, 105 genes differentially expressed in MIBC versus normal tissue, and 60 genes differentially expressed in NMIBC versus MIBC. All genes were then validated by RNA sequencing from a second cohort. A Venn diagram shows that 402/522 genes in NMIBC and 33/105 genes in MIBC were exclusively dysregulated compared to that of normal tissue, and 11/60 genes were differentially expressed only between NMIBC and MIBC (Figure 2A). To understand the biological functions of these genes, we subjected each of them to network enrichment analysis using GeneMANIA and STRING. The 402/522 differentially expressed genes in the NMIBC were strongly associated with the extracellular matrix (ECM) (false discovery rate (FDR), 1.35E-17) and extracellular structure organization (FDR, 2.98E-13) (Supplementary Table 1). The 33/105 differentially expressed genes in the MIBC were associated with mitosis (FDR, 1.72E-21) and nuclear division (FDR, 1.88E-20) (Supplementary Table 2). The 11/60 genes that were differentially expressed between NMIBC and MIBC were associated with cellular responses to zinc ions (FDR, 4.12E-11) (Supplementary Table 3). Seventy-two genes, whose functions are related to nuclear division and G2/M transition in cell mitosis, showed different expression between BC and normal tissues.

### Identification of miRNAs differentially expressed between BC tissue and normal tissue

MiRNAs were determined as shown in Figure 1. We extracted 70 miRNAs differentially expressed in NMIBC versus normal tissue, 25 miRNAs differentially expressed in MIBC versus normal tissue, and 6 miRNAs differentially expressed in NMIBC versus MIBC, all of which were validated by RNA sequencing in a second cohort. The Venn diagram shows that 58 miRNAs were exclusively deregulated in NMIBC versus normal, 17 were specifically deregulated in MIBC versus normal, and 2 miRNAs were differentially expressed only between NMIBC and MIBC (Figure 2B). By comparing our data with 190 miRNAs identified in a previously reported miRNA profiling study of BC [7], we identified 27 novel miRNAs: miR-124, miR-1260a, miR-136, miR-149, miR-191, miR-301b, miR-302a, miR-302c, miR-3065, miR-3195, miR-331, miR-335, miR-34a, miR-363, miR-371a, miR-381, miR-409, miR-4284, miR-4492, miR-4532, miR-4634, miR-483, miR-484, miR-5581, miR-5701, miR-590, and miR-654.

### NMIBC-/MIBC-specific gene-miRNA interactions

**Figure 3:**
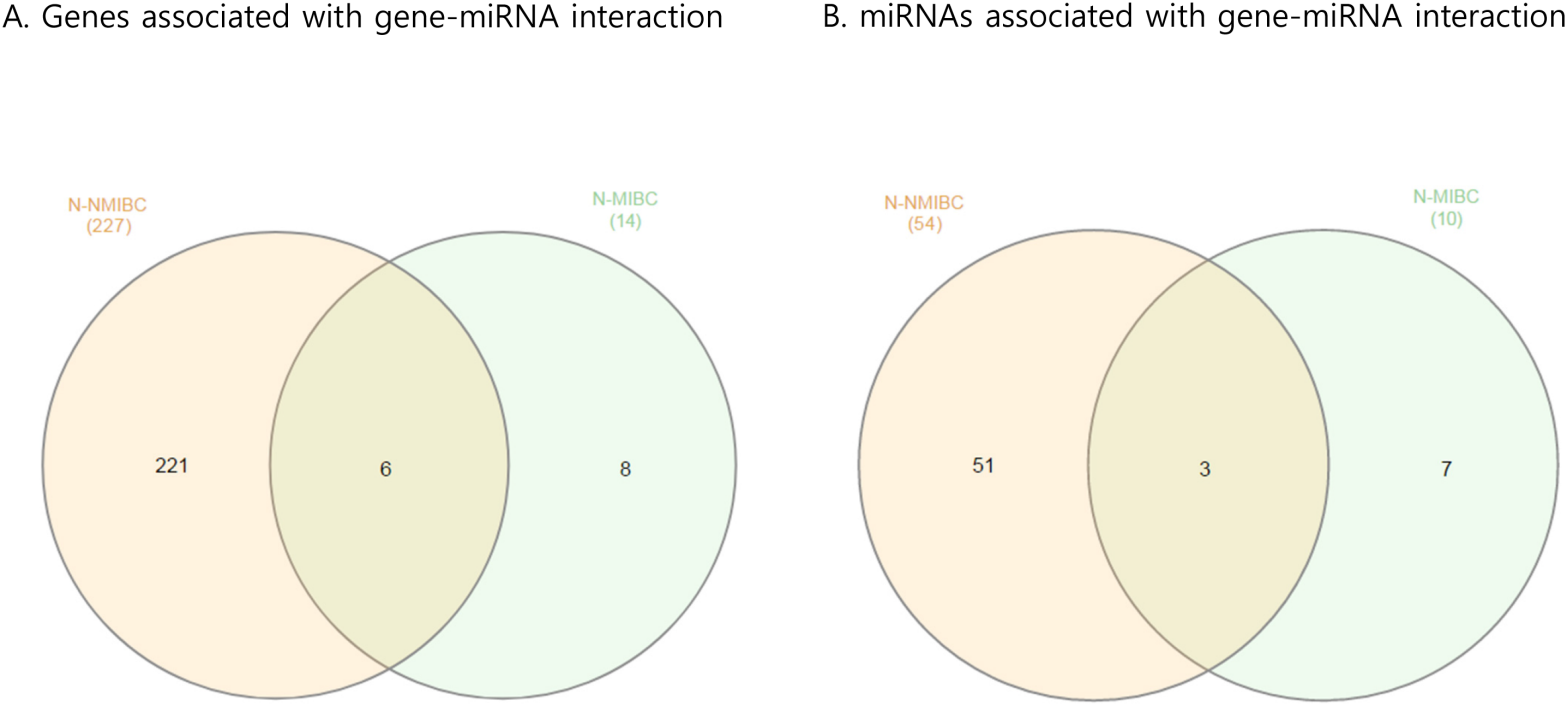
Venn diagram showing paired gene and miRNA in BC **(A)** Genes associated with gene-miRNA interactions. **(B)** miRNAs associated with gene-miRNA interactions. BC, bladder cancer; NMIBC, non-muscle invasive bladder cancer; MIBC, muscle invasive bladder cancer.

Next, we attempted to identify gene-miRNA interactions based on the validated mRNAs and miRNAs specific for NMIBC and MIBC. This analysis proceeded by two steps: first, we identified candidate gene target miRNAs based on experimentally validated datasets from the mirTarBase v6 [8] in miRWalk 2.0 [9]. Through this, we obtained 8,542 gene-miRNA interaction pairs for the 522 NMIBC genes derived from the mRNA expression profiles (NMIBC vs normal) and 1,740 gene-miRNA interaction pairs for the 105 MIBC genes (MIBC vs. normal). Next, among the gene-miRNA interaction pairs, only significantly expressed genes and miRNAs that paired with each other were selected to further analyze for differences between normal versus NMIBC and normal versus MIBC. Finally, we obtained 227 genes that paired with 54 miRNAs in NMIBC (Figure 3, Supplementary Table 4), and 14 genes that paired with 10 miRNAs in MIBC (Figure 3, Supplementary Table 5). These miRNAs were compared with190 previously reported miRNAs [10]. This enabled us to identify 14 novel miRNAs: seven (miR-1260a, miR-149, miR-191, miR-335, miR-34a, miR-484, and miR-5701) in NMIBC and seven (miR-124, miR-302c, miR-331, miR-371a, miR-4492, miR-4634, and miR-483) in MIBC (Table 1). To validate biological replication on miRNA-mRNA interacted genes, we were recruited and reanalysis GSE and the cancer genome atlas (TCGA) data with AnaltAnlyzer [11]. Finally, these 14 miRNAs showed negative correlations with 130 genes from differentially expressed analysis that were validated expression in NMIBC using GSE40355. The seven miRNAs showed negative correlations with 10 genes from differentially expressed analysis that were validated its expression in MIBC using GSE40355 [12], GSE66064 [13] and RNA sequences from TCGA (Table 1).

**Table 1:** Expression profiling of miRNA-mRNA interacted target genes in NMIBC and MIBC

a. Normal versus NMIBC														
miRNAs	miRNA Expression	Target genes	Target Gene Expression	GSE40355 Expression	miRNAs	miRNA Expression	Target genes	Target Gene Expression	GSE40355 Expression	miRNAs	miRNA Expression	Target genes	Target Gene Expression	GSE40355 Expression
miR-1260a	Up	RNF150	Down	NA	miR-335	Up	ATP8B2	Down	NA	miR-335	Up	AKAP12	Down	Down
		PRNP	Down	Down			CCL19	Down	Down			CD1D	Down	Down
miR-149	Up	CALD1	Down	Down			CD14	Down	Down			CD37	Down	Down
		FLNA	Down	Down			CD8A	Down	Down			CDKN1C	Down	Down
		GFPT2	Down	Down			CFI	Down	Down			COL21A1	Down	Down
		JUN	Down	Down			CGNL1	Down	Down			DLG2	Down	Down
		NFIX	Down	NA			COL3A1	Down	Down			F10	Down	Down
		NR2F1	Down	Down			CYGB	Down	NA			GNA14	Down	NA
miR-191	Up	EGR1	Down	Down			CYTL1	Down	Down			GPR162	Down	NA
miR-335	Up	ABCA8	Down	Down			CYYR1	Down	Down			GZMK	Down	NA
		ADAMTS8	Down	Down			DACT1	Down	Down			ITPR1	Down	Down
		ANTXR2	Down	Down			DNASE1L3	Down	Down			KLRB1	Down	Down
		AOC3	Down	Down			DOCK11	Down	NA			MAFB	Down	Down
		BIN1	Down	NA			FBLN5	Down	NA			MFGE8	Down	Down
		C1S	Down	Down			FILIP1L	Down	Down			MXRA8	Down	Down
		CDH11	Down	NA			FZD1	Down	NA			NAALAD2	Down	Down
		CLIC4	Down	Down			FZD10	Down	Down			NFIL3	Down	Down
		COLEC12	Down	NA			GABARAPL1	Down	Down			RECK	Down	Down
		COL6A1	Down	Down			GATA5	Down	NA			RERG	Down	Down
		CPE	Down	Down			GSN	Down	Down			TLR1	Down	NA
		CRISPLD2	Down	Down			HCST	Down	Down			VNN2	Down	Down
		DACH1	Down	Down			ITGB2	Down	Down	miR-34a	Up	AXIN2	Down	Down
		FBLN2	Down	NA			KIAA1644	Down	Down			AXL	Down	Down
		FGL2	Down	NA			LIFR	Down	Down			BCL2	Down	Down
		FHL1	Down	Down			LMOD1	Down	Down			CALD1	Down	Down
		FOS	Down	NA			NKG7	Down	Down			CSF1R	Down	Down
		GNA14	Down	NA			OTUD1	Down	Down			EPB41L2	Down	Down
		HLA-DRB5	Down	Down			P2RX1	Down	Down			GAS1	Down	Down
		KLF9	Down	Down			PLSCR4	Down	Down			MAP1B	Down	Down
		LMO3	Down	Down			PODN	Down	NA			RTN4	Down	NA
		LPPR4	Down	Down			PPAP2B	Down	Down			TGFBR2	Down	Up
		LTBP4	Down	Down			PRICKLE2	Down	Down			TGM2	Down	Up
		MAOB	Down	Down			PROS1	Down	Down			TPM1	Down	Down
		MGP	Down	NA			RARRES2	Down	NA			VASN	Down	NA
		NFIB	Down	Down			RASD1	Down	NA			ZEB2	Down	NA
		OLFML3	Down	Down			RHOB	Down	Down	miR-484	Up	ACTA2	Down	Down
		PDE5A	Down	NA			RORB	Down	NA			ATP1A2	Down	Down
		PDK4	Down	Down			SCARA5	Down	Down			CRTAP	Down	Down
		RNASE4	Down	Down			SCRG1	Down	NA			DSTN	Down	NA
		RNF150	Down	NA			SFRP1	Down	Down			FAM129A	Down	NA
		ROR2	Down	Down			SLC46A2	Down	Down			FLNA	Down	Down
		SERPINF1	Down	Down			SORBS2	Down	Down			KLF2	Down	Down
		SERPING1	Down	Down			TNC	Down	NA			TSC22D3	Down	Down
		SMOC2	Down	Down			TNC	Down	NA			TUBB2A	Down	Up
		SYNPO2	Down	Down			TSC22D3	Down	Down	miR-5701	Up	RUNX1T1	Down	Down
		TGFBR2	Down	Up			VASN	Down	NA					
		ZFP36L1	Down	Down			ZEB2	Down	NA					

b. Normal versus MIBC													
miRNAs	miRNA Expression	Target genes	Target Gene Expression	GSE40355 Expression	GSE66064 Expression	TCGA Expression	miRNAs	miRNA Expression	Target genes	Target Gene Expression	GSE40355 Expression	GSE66064 Expression	TCGA Expression
miR-124	Down	EZH2	Up	NA	NA	Up	miR-371a	Down	MCM10	Up	Up	Up	Up
		KIF2C	Up	NA	Up	Up			MELK	Up	Up	NA	Up
		AURKA	Up	Up	NA	Up	miR-4492	Down	CDCA8	Up	Up	Up	Up
		KIF20A	Up	NA	NA	Up	miR-4634	Down	POLQ	Up	Up	NA	Up
miR-302c	Down	MELK	Up	Up	NA	Up	miR-483	Up	FGF9	Down	Down	NA	NA
miR-331	Up	NELL2	Down	Down	NA	NA							

NA: Not available

NMIBC, non-muscle invasive bladder cancer; MIBC, muscle invasive bladder cancer.

To understand the biological functions of the genes that interacted with miRNAs, selected genes were subjected to network enrichment analysis using GeneMANIA and STRING. We found that 221 genes differentially expressed in NMIBC were strongly associated with the ECM (FDR, 4.60E-18) and extracellular structure organization (FDR, 1.72E-11). (Supplementary Table 6), whereas 8 differentially expressed genes in MIBC were involved in spindle formation (FDR, 7.53E-15) and mitosis (FDR, 2.80E-10) (Supplementary Table 7).

### The influence of transcription factors (TFs) on BC tumorigenesis

In addition to miRNAs, gene expression is also regulated by TF**s**. Therefore, we decided to investigate the relationship between TFs and miRNA-interaction genes using the transcriptional regulatory element database (TRED). Twenty-two genes (ACTA2, BCL2, C1S, CAV1, CD14, COL3A1, COL6A1, CTGF, ESR1, F3, FOS, GJA1, ITGB2, JUN, MGP, PRNP, TGFBI, TPM2, TYMS, UCHL1, VIM, and ZFP36L1) are regulated by 62 TF**s** in 36 TF families [14]. Most target genes of TFs were down-regulated in BC; an exception was the thymidylate synthetase (TYMS) gene, which is regulated by E2F4, MYC, TP53, USF1, and USF2 regulatory elements (Table 2).

**Table 2:** A lot of TFs interacted with target genes in this study

Transcription factor	Expression	Target genes	Expression	Transcription factor	Expression	Target genes	Expression	Transcription factor	Expression	Target genes	Expression
ELK1	Up	ACTA2	Down	PPARG	Up	ESR1	Down	HIF1A	Up	ITGB2	Down
ATF1	Up	BCL2	Down	RARA	Up		Down	SPI1	Up		Down
BRCA1	Up		Down	SMAD4	Up		Down	ATF1	Up	JUN	Down
CEBPA	Up		Down	SP1	Up		Down	ATF2	Up		Down
CREB1	Up		Down	SP3	Down		Down	BCL6	Up		Down
EGR1	Down		Down	TFAP2C	Up		Down	CREB1	Up		Down
ETS1	Up		Down	USF1	Up		Down	CREM	Up		Down
MYB	Up		Down	AR	Up	F3	Down	EGR1	Down		Down
MYC	Up		Down	CEBPA	Up		Down	ERG	Up		Down
NFKB1	Up		Down	CEBPB	Up		Down	ESR1	Down		Down
NFKB2	Up		Down	CEBPD	Up		Down	ESR2	Up		Down
PPARG	Up		Down	CREB1	Up		Down	ETS2	Up		Down
RARA	Up		Down	E2F4	Up		Down	JUN	Down		Down
RARB	Down		Down	EGR1	Down		Down	MYC	Up		Down
RARG	Up		Down	HIF1A	Up		Down	NFIC	Up		Down
RELA	Up		Down	JUN	Down		Down	PGR	Up		Down
STAT1	Up		Down	JUND	Up		Down	RARA	Up		Down
STAT3	Up		Down	MYB	Up		Down	RARB	Down		Down
WT1	Up		Down	NFIC	Up		Down	RARG	Up		Down
E2F1	Up	C1S	Down	NFKB1	Up		Down	REL	Up		Down
E2F2	Up+	CAV1	Down	REL	Up		Down	SMAD3	Up		Down
E2F4	Up		Down	RELA	Up		Down	SMAD4	Up		Down
ESR1	Down	CD14	Down	SP3	Down	FOS	Down	SP1	Up		Down
SP1	Up		Down	TFAP2A	Up+		Down	STAT1	Up		Down
CEBPA	Up		Down	ARNT	Up		Down	STAT3	Up		Down
CEBPB	Up		Down	ATF1	Up		Down	JUN	Down	MGP	Down
RELB	Up		Down	ATF2	Up		Down	AR	Up	PRNP	Down
SP1	Up		Down	BCL6	Up		Down	E2F4	Up		Down
SP2	Up		Down	CEBPB	Up		Down	JUN	Down		Down
SP3	Down		Down	CREB1	Up		Down	TFAP2A	Up+		Down
JUN	Down	COL3A1	Down	CREM	Up		Down	SMAD1	Up	TGFBI	Down
NFIC	Up		Down	ESR1	Down		Down	MYC	Up	TPM2	Down
SMAD1	Up		Down	ESR2	Up		Down	E2F4	Up	TYMS	Up
SMAD3	Up		Down	ETS1	Up		Down	MYC	Up		Up
AR	Up	COL6A1	Down	ETS2	Up		Down	USF1	Up		Up
SMAD3	Up		Down	ETV4	Up+		Down	USF2	Up		Up
TFAP2A	Up		Down	FLI1	Up		Down	MYB	Up	UCHL1	Down
SP1	Up	CTGF	Down	JUN	Down		Down	MYC	Up		Down
WT1	Up		Down	NFIC	Up		Down	ERG	Up	VIM	Down
AR	Up	ESR1	Down	NFKB1	Up		Down	ETV4	Up		Down
BRCA1	Up		Down	STAT1	Up		Down	LEF1	Up		Down
BRCA2	Up		Down	STAT3	Up		Down	MYB	Up		Down
ERG	Up		Down	JUN	Down	GJA1	Down	NFKB1	Up		Down
HIF1A	Up		Down	SP1	Up		Down	SP1	Up		Down
JUN	Down		Down	CEBPA	Up	ITGB2	Down	MYC	Up	ZFP36L1	Down
PAX5	Up		Down	CEBPB	Up		Down				

TF, transcription factor; BC, bladder cancer

## DISCUSSION

In this study, we used two different methods to identify global miRNA-mRNA interactions and their enriched functional networks that are specific to NMIBC and MIBC. Microarrays were used to obtain differential expression profiles, while RNA-Seq was used for experimental validation of both miRNA and mRNA levels. Additionally, those gene expression pattern were validated using another cohort which were previously reported GSE and TCGA [11–13]. These results showed that expression pattern of gene-miRNA interacted genes were conserved in NMIBC and MIBC on tumorigenesis. We uncovered detailed patterns of miRNA and gene expression, allowing us to propose molecular markers for specific subtypes of BC. The 402 genes differentially expressed in NMIBC were related to ECM function, platelet activation, and multicellular organismal metabolism. Moreover, we found that the miRNAs interacted with the 221 genes in NMIBC were mainly related to the ECM, organization of extracellular structure, and muscle contraction. In contrast, the 33 genes differentially expressed in MIBC were mainly related to mitosis, antigen processing and presentation via MHC class II, and G2/M transition. In addition, we found that 8 miRNA-interaction genes in MIBC were related to spindle formation, mitosis, and nuclear division.

The results of gene-network analysis of gene-miRNA pairs revealed that ADAMTS8, COL21A1, COL3A1, COL6A1, COL6A2, COL18A1, FBLN2, FBLN5, F3, LTBP4, MFGE8, MGP, PARRES2, SERPINF1, SFRP1, SNCA, TGFBI, TGFB3, TNC, and VEGFA genes, which were down-regulated in NMIBC, are involved in the formation or function of the ECM, including the release of new growth factors and ECM molecules. The ECM serves as a structural scaffold that provides the support necessary to maintain tissue integrity and sustainability [15]. Therefore, ECM regulation is important for the generation of new tissue structures, as well as maintaining the architecture and homeostasis of adult tissues [15]. In addition to our discovery of a potential panel of genes for BC, the genes we found that modulate smooth muscular contraction (ACTA2, ATP1A2, CALD1, CAV1, EDNRA, GJA1, LMOD1, MYH11, MYLK, TPM1, TPM2, and VIM) could represent potential therapeutic targets for the treatment of diseases related to bladder contraction [15].

Cell cycle-related genes (AURKA, CDCA8, KIF20A, and KIF2C) were overexpressed in MIBC compared to NMIBC. Moreover, genes that play a role in cell cycle division are over-expressed in BC when compared with normal tissues. Therefore, since cell division, chromatin replication, and chromosome segregation are key targets for drug development, miRNA-targeted genes involved in nuclear division and G2/M transition may be candidate targets for drugs designed to treat BC [16].

Previously published studies propose 440 up- or down-regulated miRNAs as BC biomarkers, which comprise 190 union miRNAs. Among them, miR-145 was down-regulated in multiple different types of cancer tissue, helping to maintain the differentiation status of smooth muscle cells. This observation was reported consistently by 11 other groups [7], suggesting that miR-145 is likely to be a critical factor in BC tumorigenesis. Finally, our comprehensive analyses identified 14 novel miRNAs specific to BC (miR-124, miR-1260a, miR-149, miR-191, miR-302c, miR-331, miR-335, miR-34a, miR-371a, miR-4492, miR-4634, miR-483, miR-484, and miR-5701).

Members of the miR-183 cluster are located within a 5 kb region on human chromosome 7q32.2, and are transcribed in the same direction (from telomere to centromere) [17]. Previous studies have shown that the miR-183 cluster was abnormally expressed in a variety of tumors and could be directly involved in human cancers [18]. Notably, miR-183-3p, miR-182-5p, and miR-96-5p have oncogenic functions in BC [19, 20], and we found here that these miRNAs were over-expressed in both NMIBC and MIBC. In particular, the miR-183 cluster regulates genes that overlap in both NMIBC and MIBC, i.e., DKK3 (a target of miR-183-5p), FGF9 (a target of miR-182-5p), and monooxygenase, DBH-Like 1 (MOXD1, a target of miR-96-5p), suggesting that these miRNAs play important roles in bladder tumorigenesis. Our present study is also one of the first to reveal that MOXD1 is regulated by miR-96-5p in BC tissue. MOXD1 encodes a member of the copper monooxygenase/dopamine/β-hydroxylase family, which is localized to the endoplasmic reticulum. Members of this protein family are mainly involved in biosynthesis of neurotransmitters and hormones.

Furthermore, several lines of evidence indicate that miR-200b and miR-200c are over-expressed to a greater extent in NMIBC than in MIBC, consistent with previous studies [21]. The miR-200 family is thought to play an essential role in tumor suppression by inhibiting the epithelial-mesenchymal transition (EMT), an initiating step of metastasis [22]; because cells lose adhesion during the EMT, the rate of cell mortality is elevated during this process. In particular, miR-200 promotes the final step of metastasis in which migrating cancer cells undergo EMT during colonization of distant tissues [23]. Interestingly, our data shows that miR-200b and miR-200c were more highly expressed in NMIBC than in MIBC. Moreover, our study is the first to report the BCL2 (a target of miR-200b-3p and miR-200c-3p), CDH11 (a target of miR-200c-3p), FBLN5 (a target of miR-200c-3p), and TIMP2 (a target of miR-200c-3p) interaction pairs in NMIBC (Supplementary Table 4a).

Through analysis of the gene-miRNA pairs after linking the expression profiles of TFs and miRNAs to their target genes, we found that the expression of almost all TFs was moderately or severely repressed while miRNAs were over-expressed in BC tissues versus normal tissues. The exceptions to this general trend were E2F2, TFAP2A, and ETV4; TFs that were over-expressed in BC tissue. These results suggest that these TFs and miRNAs may regulate target genes with oncogenic properties in BC tumorigenesis. While almost all of the target genes in the gene-miRNA pairs showed lower expression, the TYMS gene interestingly showed over-expression, indicating possible oncogenic properties. This suggests that miR-99a-5p, which interacts with TYMS, and TFs E2F4, MYC, USF1, and USF2 regulate the expression of the TYMS gene. Further work is needed to gain a better understanding of how TFs and miRNAs act as both oncogenes and tumor suppressors. Advancements could potentially lead to the development of therapeutic targets from the expression profiling data of TFs, miRNAs, and the target genes in BC.

In summary, our validated data obtained from NGS-based experiments, combined with comprehensive and unbiased computational analyses of published data, identified 14 novel miRNAs in BC. Furthermore, genes differentially expressed in NMIBC were associated with the ECM, muscle contraction, and nuclear division. Gene enrichment analysis of the gene-miRNA pairs also suggest that the organization of the ECM is important in NMIBC, whereas DNA replication and G2/M transition are important in MIBC. This report provides the first evidence for novel gene-miRNA interaction pairs involving 227 genes and 54 miRNAs in NMIBC and 14 genes and 10 miRNAs in MIBC.

The promising findings from this study may lead to potentially novel diagnostic and therapeutic interventions in BC and other cancers.

## MATERIALS AND METHODS

### Patients

The study cohort included 234 patients histologically diagnosed with bladder urothelial carcinoma and 83 individuals without BC. Tumor levels were assessed according to standard criteria [3]. The BC samples were further subcategorized into two groups, NMIBC and MIBC. NMIBC patients, who were usually treated with TUR, were periodically assessed by cystoscopy and urinary cytology every 3 months for the first 2 years, every 6 months for the next 3 years, and annually thereafter. Patients with MIBC underwent radical cystectomy and complete pelvic lymph node dissection. These patients were also subjected to urinary diversion, including creation of a conduit, continent cutaneous reservoir, and orthotopic ileal neobladder. Moreover, MIBC patients with pT3/pT4/lymph node–positive disease were subjected to four to six cycles of cisplatin-based adjuvant chemotherapy. These MIBC patients were subjected to physical examination, urine cytology, serum chemistry, chest x-ray, and abdominal and pelvic computerized tomography every 3 months for the first 2 years, every 6 months for the following 2 years, and then annually thereafter. Clinical data was assessed for all patients retrospectively. The mean follow-up of these patients was 71 months (median 61 months; range, 15–115 months). Normal muscosae was collected far from tumors and real normal mucosae from benign diseases. All specimens were rapidly frozen in liquid nitrogen and stored at -80°C until use. Collection and analysis of samples was approved by the Institutional Review Board of Chungbuk National University (IRB approval number 2006-01-001 and GR2010-12-010), and informed consent was obtained from each subject.

### Datasets

High-throughput molecular datasets were generated for miRNAs using two different technologies. Microarray and RNA sequencing mRNA data were generated by RNA sequencing. The analysis also included microarray datasets published previously by our group (Gene Expression Omnibus accession number GSE13507) [24]. Sample sizes are provided in Figure 1.

### Library preparation and data generation

#### RNA extraction

Total RNA was isolated from the indicated tissues using the TRIzol reagent (Life Technologies, Carlsbad, CA) and purification was performed using phenol based extraction methods according to the manufacturer’s instructions. The RNA concentration was determined using NanoDrop ND-1000 spectrometer and the RNA integrity number was evaluated on a 2100 Bioanalyzer (Agilent, Santa Clara, CA) using the RNA 6000 Nano Kit (Agilent, Santa Clara, CA).

#### miRNA microarrays

Total RNA (100 ng) from each sample was dephosphorylated, 3' end–labeled with Cy3-pCp, purified on Micro Bio-Spin columns, dried, and hybridized using the miRNA Microarray System labeling kit ( Illumina, San Diego, CA) and the Agilent Human miRNA Microarray Release 16.0 platform, which contains 1,205 human and 144 viral miRNAs [25]. The protocol used to generate microarray gene expression datasets is provided in reference [26].

#### Stranded mRNA library construction

mRNA sequencing libraries were prepared using the TruSeq Stranded mRNA Sample Preparation Kit (RS-122-2101) (Illumina, San Diego, CA). Oligo dT attached magnetic beads were used to purify poly-A–containing mRNA from 1 μg of total RNA. Next, the purified mRNA was disrupted into short fragments, and first-strand cDNAs were synthesized using SuperScript II reverse transcriptase (Invitrogen, Carlsbad, CA) and random hexamers. cDNA with adapters ligated to both ends were enriched by PCR. cDNA library size and quality were evaluated electrophoretically using the Agilent DNA 1000 Kit (part # 5067-1504) on a 2100 BioAnalyzer. Subsequently, the libraries were sequenced on an Illumina HiSeq 2500. Image analysis was performed using the HiSeq control software version 2.2.58. Raw data were processed and base calling was performed using the standard Illumina pipeline (CASAVA version 1.8.2 and RTA version 1.18.64).

#### Small RNA library construction

Small RNA sequencing libraries were constructed using the TruSeq Small RNA Sample Preparation protocol (RS-200-0012) (Illumina, San Diego, CA). Illumina adapters were directly and specifically ligated to microRNA molecules with a 3'-hydroxyl group and a 5'-phosphate. The quality and size distribution of the adapter-ligated RNAs and amplified libraries were confirmed using the High Sensitivity DNA Analysis Kit (Cat. #5067-4626) (Agilent, Santa Clara, CA). Libraries were quantitated using the Library Quantification Kit for NGS (KK4824) (Kapa Biosystems, Wilmington, MA). Subsequently, libraries were sequenced on an Illumina HiSeq 2500. Real-time image analysis and base calling were performed on the instrument using the HiSeq Sequencing Control Software version 2.2.58. CASAVA software version 1.8.2 and RTA version 1.18.64 were used for de-multiplexing and generation of FASTQ sequence files.

### Data analysis

#### Microarrays

The Robust Multiarray Average, in the R package [27], was used to perform global correction, quantile normalization, and median Polish summarization. *P*-values (t test) were calculated from bead mRNA signal intensities [27].

#### mRNA sequencing

Total sequencing reads were subjected to preprocessing as follows: adapter trimming was performed using cutadapt with default parameters, and quality trimming (Q30) was performed using FastQC with default parameters. Processed reads were mapped to the human reference genome (Ensembl 72 [GRCh37: hg19]) using tophat and cufflink with default parameters [28]. Fragments Per Kilobase of exon per million fragments Mapped (FPKM) values were normalized and quantitated using R package Tag Count Comparison (TCC) [29] to determine statistical significance (e.g., P and Q values) and differential expression (e.g., -fold changes).

#### miRNA sequencing

Total sequencing reads were subjected to preprocessing: adapter trimming, quality trimming, size selection (17–24 nt), and clustering using mirDeep2 with default parameters [30]. The preprocessed cluster representatives were mapped to the human reference genome (Ensembl 72 [GRCh37; hg19]), and miRNA regions were mined with respect to miRBase reference co-ordinates. Total read counts were subjected to Trimmed mean m-values normalization and quantitation using R package edgeR, and statistical significance (e.g., P and Q values) and differential expression (e.g. -fold changes) were determined.

#### Integrated analysis of differentially expressed miRNAs and mRNAs

For data obtained using each technology, we used different cut-offs to identify differentially expressed mRNAs and miRNAs. Initially, thresholds for p-values (*P* ≤ 0.05) and -fold changes (log_2_FC ≥ 1.0) were applied to the microarray dataset. Thresholds for the RNA-sequencing datasets were as follows: mRNAs (FPKM ≥ 0.3, *P* ≤ 0.05, FDR ≤ 0.05, and log_2_FC ≥ 1.0) and miRNAs (read count ≥ 10, *P* ≤ 0.05, FDR ≤ 0.05, and log_2_FC ≥ 1.0). Finally, mRNAs common to the microarray dataset and mRNA-Seq datasets were selected as validated mRNAs. MiRNAs common to the microarray dataset and miRNA-Seq datasets were selected as validated miRNAs. The selected mRNAs and miRNAs were paired using validated target datasets and according to inverse expression relationships (i.e., miRNAs whose level increased as that of their target decreased and *vice versa*).

#### Analysis of the relation between transcription factors (TFs) and miRNA-interacting genes

TRED was used to identify the TFs related to the miRNA targeting genes in BC. There are curated 36 cancer-related TF families in TRED [14]. And we compared the miRNA-mRNA interacted pairs with the known TF families.

#### Pathway enrichment analysis

Pathway enrichment analysis was performed by submitting selected miRNAs to DIANA mirPath V3.0 [31]. Significantly correlated and enriched pathways were identified by calculating significant p-values using Fisher’s meta-analysis method for specific miRNAs. Finally, the list of genes was examined using GeneMANIA [32] to identify interactive functional networks from existing databases.

## SUPPLEMENTARY MATERIALS TABLES





## Abbreviations

BC: Bladder cancer
ECM: Extracellular matrix
ETM: Epithelial-mesenchymal transition
FDR: False discovery rate
MIBC: Muscle invasive bladder cancer
miRNA: microRNA
MOXD1: Monooxygenase DBH-like 1
NMIBC: Non-muscle invasive bladder cancer
TCC: Tag count comparison
TCGA: The cancer genome atlas
TF: Transcription factor
TRED: Transcriptional regulatory element database
TUR: Transurethral resection
TYMS: Thymidylate synthetaset
