# Supplementary material for: Identification of differentially expressed miRNAs and miRNA-targeted genes in bladder cancer
Source: Oncotarget. 2018 Feb 7;9(45):27656–66. doi: 10.18632/oncotarget.24441 (PMC6021253; doi:10.18632/oncotarget.24441)
Supplement: Supplementary file 1 [file oncotarget-09-27656-s001.pdf]

Identification of differentially expressed miRNAs and miRNA-targeted genes in bladder cancer

SUPPLEMENTARY MATERIALS

Supplementary Table 1: Biological functions of 402 genes that were differentially expressed between NMIBC and normal tissues

Biological Functions	FDR	Genes in network
Extracellular matrix	1.35E-17	35
Extracellular structure organization	2.98E-13	35
Extracellular matrix organization	2.98E-13	35
Platelet alpha granule	1.46E-05	12
Platelet activation	1.46E-05	21
Multicellular organismal metabolic process	3.61E-05	14
Extracellular matrix disassembly	6.17E-05	15
Collagen metabolic process	6.17E-05	13
Platelet alpha granule lumen	7.54E-05	10
Endoplasmic reticulum lumen	7.54E-05	16
Multicellular organismal macromolecule metabolic process	7.54E-05	13
Proteinaceous extracellular matrix	0.000103	14

NMIBC, non-muscle invasive bladder cancer

Supplementary Table 2: Biological functions of 33 genes that were differentially expressed between MIBC and normal tissues

Biological Functions	FDR	Genes in network
Mitosis	1.72E-21	19
Nuclear division	1.88E-20	20
Organelle fission	8.27E-20	20
Spindle	5.72E-15	14
Condensed chromosome kinetochore	2.21E-12	8
Chromosome segregation	2.88E-12	12
Condensed chromosome, centromeric region	7.66E-12	8
Kinetochore	1.49E-11	10
Regulation of cell division	1.70E-11	12
Microtubule cytoskeleton organization	3.66E-11	13
Sister chromatid segregation	3.66E-11	9
Condensed chromosome	4.78E-11	10

MIBC, muscle invasive bladder cancer.

Supplementary Table 3: Biological functions of 11 genes that were differentially expressed between NMIBC and MIBC tissues

Biological Functions	FDR	Genes in network
Cellular response to zinc ion	4.12E-11	6
Response to zinc ion	4.88E-10	6
Response to transition metal nanoparticle	2.41E-08	6
Cellular response to metal ion	5.77E-07	6
Cellular response to inorganic substance	7.62E-07	6
Response to cadmium ion	5.72E-06	4
Response to metal ion	3.61E-05	6
Negative regulation of growth	1.22E-04	6
Response to inorganic substance	3.00E-04	6
Transition metal ion binding	7.32E-04	7
Zinc ion binding	2.25E-03	6
Perinuclear region of cytoplasm	7.07E-03	6

NMIBC, non-muscle invasive bladder cancer; MIBC, muscle invasive bladder cancer.

Supplementary Table 4: List of negatively correlated pairs of 227 genes and 54 miRNAs in NMIBC

See Supplementary File 1

Supplementary Table 5: List of negatively correlated pairs of 14 genes and 10 miRNAs in MIBC

Gene	miRNA	Gene expression	miRNA expression
DKK3	miR-183-5p	down-regulated	up-regulated
FGF9	miR-483-3p	down-regulated	up-regulated
ITGA8	miR-183-5p	down-regulated	up-regulated
MGLL	miR-7-5p	down-regulated	up-regulated
MOXD1	miR-96-5p	down-regulated	up-regulated
NELL2	miR-331-3p	down-regulated	up-regulated
AURKA	miR-124-3p	up-regulated	down-regulated
CDCA8	miR-4492	up-regulated	down-regulated
EZH2	miR-124-3p	up-regulated	down-regulated
KIF20A	miR-124-3p	up-regulated	down-regulated
KIF2C	miR-124-3p	up-regulated	down-regulated
MCM10	miR-371a-5p	up-regulated	down-regulated
MELK	miR-371a-5p	up-regulated	down-regulated
	miR-302c-3p	up-regulated	down-regulated
POLQ	miR-4634	up-regulated	down-regulated

MIBC, muscle invasive bladder cancer.

Supplementary Table 6: Biological functions of 217 genes from gene-miRNA pairs that were differentially expressed between NMIBC and normal tissues

Biological Functions	FDR	Genes in network
Extracellular matrix	4.60E-18	29
Extracellular structure organization	1.72E-11	26
Extracellular matrix organization	1.72E-11	26
Proteinaceous extracellular matrix	4.58E-06	13
Muscle contraction	4.53E-05	15
Extracellular matrix disassembly	8.44E-05	12
Muscle system process	1.47E-04	15
Muscle structure development	1.47E-04	16
Muscle organ development	4.57E-04	13
Actin cytoskeleton	6.63E-04	16
Transmembrane receptor protein S	6.63E-04	16
Serine/threonine kinase signaling pathway	8.59E-04	14

NMIBC, non-muscle invasive bladder cancer.

Supplementary Table 7: Biological functions of 8 genes from gene-miRNA pairs that were differentially expressed between MIBC and normal tissues

Biological Functions	FDR	Genes in network
Spindle	7.53E-15	12
Mitosis	2.80E-10	10
Nuclear division	2.80E-10	11
Organelle fission	4.39E-10	11
Microtubule cytoskeleton organization	1.15E-09	10
Midbody	6.20E-08	7
Microtubule	7.79E-08	8
Regulation of cell division	8.44E-08	8
Microtubule-based movement	1.40E-07	7
Chromosome, centromeric region	1.46E-07	7
Chromosome segregation	8.79E-07	7
Spindle organization	9.47E-07	6

MIBC, muscle invasive bladder cancer.
